# Elevated serum transaminase activities were associated with increased serum levels of iron regulatory hormone hepcidin and hyperferritinemia risk

**DOI:** 10.1038/srep13106

**Published:** 2015-08-20

**Authors:** Peng An, Hao Wang, Qian Wu, Xin Guo, Aimin Wu, Zhou Zhang, Di Zhang, Xiaochen Xu, Qianyun Mao, Xiaoyun Shen, Lihong Zhang, Zhiqi Xiong, Lin He, Yun Liu, Junxia Min, Daizhan Zhou, Fudi Wang

**Affiliations:** 1 State Key Laboratory of Bioreactor Engineering, East China University of Science and Technology, Shanghai 200237, China; 2 Key Laboratory of Nutrition and Metabolism, Institute for Nutritional Sciences, Shanghai Institutes for Biological Sciences, Chinese Academy of Sciences, University of Chinese Academy of Sciences, Shanghai 200031, China; 3 Department of Nutrition, Research Center for Nutrition and Health, Institute of Nutrition and Food Safety, School of Public Health, School of Medicine, Collaborative Innovation Center for Diagnosis and Treatment of Infectious Diseases, Zhejiang University, Hangzhou 310058, China; 4 Bio-X Institutes, Key Laboratory for the Genetics of Developmental and Neuropsychiatric Disorders (Ministry of Education), Shanghai Jiao Tong University, Shanghai 200032, P.R. China; 5 Institutes of Biomedical Sciences, Fudan University, Shanghai 200032, China; 6 Key Laboratory of Molecular Medicine, The Ministry of Education, Department of Biochemistry and Molecular Biology, Fudan University Shanghai Medical College, Shanghai 200032, P.R. China; 7 The first affiliated Hospital, Institute for Translational Medicine, School of Medicine, Collaborative Innovation Center for Diagnosis and Treatment of Infectious Diseases, Zhejiang University, Hangzhou 310058, China; 8 Department of Nutrition, Research Center for Nutrition and Health, College of Public Health, Zhengzhou University, Zhengzhou 450001, China

## Abstract

Iron imbalance is a feature of liver damage. However, the biological correlation of serum hepcidin, a key regulator of iron homeostasis, with liver malfunction is undefined. To this end, we piloted the Chinese population studies to address whether hepcidin is linked to liver functionality. The serum hepcidin, ferritin, alanine transaminase, aspartate transaminase, gamma-glutamyltransferase and bilirubin were examined in two independent Chinese cohorts consisted of 3455 individuals. After adjustment for sex, age, body mass index, smoking habits, drinking categories and diabetic status, a positive association between hepcidin and alanine transaminase (ALT) (beta = 0.18 ± 0.01, *P* < 0.0001) was discovered using linear regression in a cohort consisting of 1813 individuals. This association was then validated in the second independent cohort of 1642 individuals (beta = 0.08 ± 0.02, *P* < 0.0001). Furthermore, consistent with cohort study, by applying both CCl_4_ and lipopolysaccharide induced mouse liver injury models, at least 2-fold elevations in hepcidin expression, serum ALT and inflammatory cytokine IL-6 were discovered during the initiation stage of liver injury. Our findings suggest that increased serum hepcidin may reflect a protective response to the iron status and elevated serum cytokines during liver injury. Additional studies are warranted to validate these findings and test their potential clinical relevance in patients.

Iron disorder is one of the important causes of liver damage^1^,^2^. The liver contributes to the maintenance of iron homeostasis through two major mechanisms: 1) iron storage, and 2) secretion of the regulatory hormone hepcidin. Dietary iron is absorbed by intestinal epithelium and then transported into circulation through the actions of ferroportin. Absorbed iron is quickly bound to iron-binding protein, transferrin. Transferrin-bound iron is delivered to erythroid precursor cells and other body cells. Another important source of iron is iron recycling from senescent erythrocytes by macrophages. Excess plasma iron will be stored in hepatocytes in the form of ferritin.

Liver-derived hepcidin is the central regulatory hormone controlling iron absorption from the small intestine and iron recycling from senescent erythrocytes. Hepcidin can trigger the internalization of iron export protein ferroportin to block iron release from small intestine, macrophages and other body cells^3^. Mutations in genes involved in the hepcidin regulation will cause hemochromatosis in humans^4^, resulting in dysregulated dietary iron absorption and iron accumulation in liver, pancreas, heart and other organs. Iron overload may progress to liver fibrosis, cirrhosis, and hepatocellular carcinoma^5^. As a result, there is an elevation of serum transaminases in most hemochromatosis patients^6^ and in mouse models of iron-induced liver injury^7^.

In addition, abnormal iron homeostasis and hepcidin dysregulation are the common features in many liver diseases. For example, increasing evidences suggest that the suppression of hepcidin in chronic hepatitis C patients aggravates the iron burden, which exacerbates liver injury^8^,^9^,^10^,^11^. Hepcidin expression has also been reported to be even more suppressed in hepatocellular carcinoma patients than in those with chronic hepatitis^12^. To understand the relationship between hepcidin and liver function parameters, we analyzed serum hepcidin concentrations in a cohort consisting of 1813 individuals^13^ and another independent cohort consisting of 1642 individuals.

## Methods

### Study Cohorts

Participants in Corhort 1 and Cohort 2 in this study were recruited from Shanghai, China. This study is a cross-sectional study using the baseline data from two cohorts. The samples of Cohort 1 were selected from our previous study^13^, including 1813 individuals recruited in 2006. Cohort 2 was another independent cohort including 1642 individuals recruited from Shanghai in 2008. Same enrollment criteria have been applied in Cohort 2. Subjects were eligible for enrollment if: 1) they were stable residents for at least 20 years in the area; 2) they were free of severe psychological disorders, physical disabilities, or cancer and had no history of stroke, coronary heart disease, Alzheimer’s disease, or dementia; and 3) they had not been currently diagnosed with tuberculosis or other communicable diseases. Home interviews were conducted by trained physicians or public health workers from the Pudong, Baoshan and Jinshan Centers for Disease Control and Prevention and community hospitals. For all individuals, height, weight, hip and waist circumference, and blood pressure were measured by trained medical professionals using a standardized protocol. Fasting blood specimens were collected using vacuum negative pressure tubes. Each serum was divided into several individual tubes and all the samples were stored at −80 °C. We provided basic features of the population samples in Table 1. This study was in accordance with the ethical guidelines of the Declaration of Helsinki. Informed consent was obtained from all study subjects and the ethics committee of the Shanghai Institute for Biological Sciences approved this study.

### Serum parameters

Both Cohort 1 and Cohort 2 applied the same method for measuring related serum parameters. Serum hepcidin was measured according to manufacturer’s instruction, using a commercially available human hepcidin ELISA kit (DRG Diagnostics GmbH, Marburg, Germany) that was based on a competitive binding method (detect range: 0.9–140 ng/mL). The 5%–95% range of serum hepcidin levels in our study was 9.02–71.82 ng/mL in male and 9.07–70.59 ng/mL in female. Serum ferritin was measured using a human ferritin ELISA kit (DRG Diagnostics GmbH, Marburg, Germany). Male individual with serum ferritin >300 ng/mL and female with serum ferritin >200 ng/mL were classified as hyperferritinemia. Hemoglobin and C-reactive protein (CRP) were measured enzymatically according to standard methods with a modular P800 model auto-analyzer (Roche Diagnostics GmbH, Mannheim, Germany) with purchased reagents (Roche Diagnostics GmbH, Mannheim, Germany). ALT, AST, GGT, TBIL, DBIL, and IBIL were measured enzymatically according to standard methods using 7180 Clinical Analyzer (Hitachi High-Technologies Corp., Tokyo, Japan) with reagents (Wako Pure Chemical Industries, Tokyo, Japan).

### Animals and treatment

Animals were 9 weeks old C57BL/6 male mice provided with a standard rodent laboratory diet (SLAC Laboratory Animal Co. Ltd., China) under specific pathogen-free conditions. Mice were randomly divided into five groups (n = 5 each) and then were intraperitoneally injected with CCl_4_ (6 μL/mouse, 2% in olive oil). The 0 h control group was injected with olive oil only. Mice were killed at specific times (0, 6, 12, 24 and 48 h post-injection), and blood and tissues were collected. For lipopolysaccharide injection, mice were randomly divided into five groups (n = 5), each mouse was intra-peritoneally injected with lipopolysaccharide (5 mg/kg body weight; Santa Cruz Biotechnology) or the same volume of phosphate-buffered saline, whole blood and tissues were collected at specified time points (0, 3, 12, 24 and 48 h post-injection). Experimental protocols were carried out in accordance with the approved guidelines of Institutional Animal Care and Use Committee of the Institute for Nutritional Sciences, Shanghai Institutes for Biological Sciences, and Chinese Academy of Sciences.

### Mouse serum parameters and iron analysis

Serum iron and transferrin saturation were measured using the Iron/TIBC reagent set (Pointe Scientific, US) according to the manufacturer’s instructions. Serum ALT and AST were determined using assay kits (Shensuoyoufu, China). Serum ferritin was quantitatively determined using mouse ferritin ELISA kit (ALPCO Diagnostics, US) and serum IL-6 was detected using mouse IL-6 ELISA kit (R&D systems, US). Measurement of tissue non-heme iron was performed using a ferrozine-based method described before^14^.

### Mouse tissue RNA extraction and quantitative real-time PCR

Mouse tissue RNA extraction was accomplished using Trizol (Invitrogen) and quantitative real-time PCR was performed using SYBR Green Supermix (Bio-Rad) on the CFX96 System (Bio-Rad). Detailed procedures were described previously^14^. Primer sequences in this study were: *β-actin* forward 5′-AAATCGTGCGTGACATCAAAGA-3′, *β-actin* reverse 5′-GCCATCTCCTGCTCGAAGTC-3′, *Bmp6* forward 5′- AACCTTTCTTATCAGCATTTACCA-3′, *Bmp6* reverse 5′- GTGTCCAACAAAAATAGGTCAGAG-3′, *Hamp1* forward 5′- GCACCACCTATCTCCATCAACA-3′, *Hamp1* reverse 5′- TTCTTCCCCGTGCAAAGG-3′.

### Statistical analysis

Data in tables are baseline data and presented as mean (SD) or median (interquartile range). *P* values in Table 1 are the comparison results between females and males using Wilcoxon rank-sum test for parameters or chi-squared test for count data. Linear regression model (hepcidin as an independent variable) was used to analyze the relationship between serum hepcidin and other serum parameters. Parameters with skewed distributions, including serum hepcidin, ferritin, CRP, ALT, AST, and GGT, were natural log-transformed before entering into regression analysis. But betas and standard errors (s.e.) were presented in non-transformed values. Logistic regression was used to determine the association of transaminase concentrations with hyperferritinemia risk (serum ferritin >200 ng/mL in women and 300 ng/mL in men). Odds ratio (OR) was calculated from the exponentiated beta coefficient (e^beta^) of logistic regression. Results in Cohort 1 were adjusted for sex, age, body mass index, smoking habits, drinking categories and diabetic status. Results in Cohort 2 were adjusted for sex, age, body mass index and diabetic status. For boxplot, band inside box indicates median; bottom of the box indicates first quartile; top of the box indicates 3rd quartile; lower whisker indicates 1.5 interquartile range below the first quartile; upper whisker indicates 1.5 interquartile range above the third quartile. Jonckheere-Terpstra test was used to obtain the trend *P* values of serum parameters according to ALT quartiles. In animal experiments, analysis of variance (ANOVA) with following by Tukey post hoc test was used to compare group means. When necessary, data with unequal variance were log-transformed to meet the assumption of homogeneity of variance (Bartlett’s test). Significance was considered at *P* < 0.05. Statistical analyses were performed using R (http://www.r-project.org/). Jonckheere-Terpstra test was performed using clinfun package of R (http://cran.r-project.org/web/packages/clinfun).

## Results

### Hepcidin showed positive association with ALT in the Cohort 1

Serum hepcidin levels were measured in 1813 participants from a previously reported cohort (the Cohort 1) (Table 1)^13^. Serum parameters in liver function tests and body iron parameters were measured (Table 1). Firstly, the relationship between serum hepcidin and other iron parameters was determined using linear regression model. Hepcidin was significantly associated with hemoglobin concentration (beta = 0.01 ± 0.003, *P* = 0.002) and serum ferritin level (beta = 0.42 ± 0.18, *P* = 0.03) after adjustment for covariates including sex, age, body mass index (BMI), smoking habits, drinking categories and diabetic status (Table 2). Among the parameters in liver function tests, alanine transaminase (ALT) was found to be significantly associated with serum hepcidin (beta = 0.18 ± 0.01, *P* < 0.0001). Exclusion of individuals with type II diabetes did not substantially change the significance of the results. Serum hepcidin level and transaminase concentrations in our study were comparable with other studies^15^,^16^.

### Hepcidin was positively associated with ALT in the Cohort 2

To confirm the association of hepcidin with ALT in the Cohort 1, serum hepcidin was measured in blood samples from another independent Chinese cohort consisting of 1642 participants (Cohort 2). The cohort size and the mean age of Cohort 2 were similar to Cohort 1. Detailed characteristics of the Cohort 2 are listed in Table 1. The association between ALT and serum hepcidin was significant in the Cohort 2 (beta = 0.08 ± 0.02, *P* < 0.0001), as was aspartate transaminase (AST) (beta = 0.04 ± 0.01, *P* < 0.0001) using linear regression model adjusted for age, sex, body mass index (BMI) and diabetic status (Table 2). For iron parameters, serum ferritin was also found to be associated with serum hepcidin (beta = 0.31 ± 0.21, *P* = 0.03).

### Increased transaminases were closely associated with elevated iron parameters

**Figure 1:**
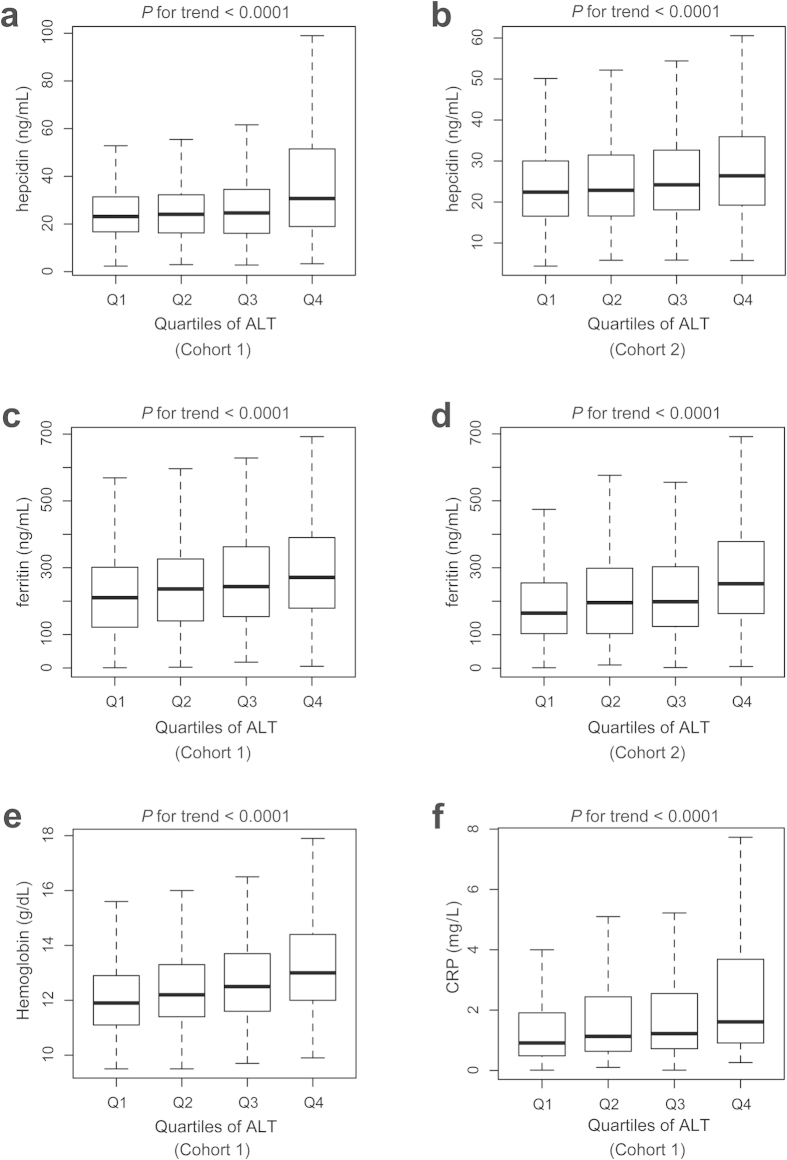
Serum iron parameter levels according to quartile of ALT. Serum hepcidin levels significantly increased according to quartile of ALT in Cohort 1 (**a**) and Cohort 2 (**b**) The same trends were also found in serum ferritin levels according to ALT quartiles in Cohort 1 (**c**) and Cohort 2. (**d**) In Cohort 1, hemoglobin concentration (**e**) and C-reactive protein (CRP) levels (**f**) were also significantly elevated with increasing quartiles of ALT. The quartiles of ALT in Cohort 1 are 8, 13 and 19 IU/L; medians of each quartile are 6, 12, 16 and 27 IU/L (n = 379–380 for each quartile). The quartiles of ALT in Cohort 2 are 10, 14 and 18 IU/L; medians of each quartile are 7, 12, 15 and 26 IU/L (n = 410–411 for each quartile). Band inside box, median; bottom of the box, first quartile; top of the box, 3rd quartile; lower whisker, 1.5 interquartile range below the first quartile; upper whisker, 1.5 interquartile range above the third quartile.

Further analysis revealed that despite the association with serum hepcidin levels (Fig. 1a,b), increased serum transaminase levels were also associated with elevated serum ferritin in two independent cohorts (Fig. 1c,d). Hemoglobin and C-reactive protein (CRP) concentrations were also elevated with the increasing quartiles of ALT in Cohort 1 (Fig. 1e,f). AST and gamma-glutamyltransferase (GGT) also showed significant associations with increased serum iron parameters and hyperferritinemia risk (Table 3).

### Hepcidin, iron status and transaminases in induced mouse liver injury models

**Figure 2:**
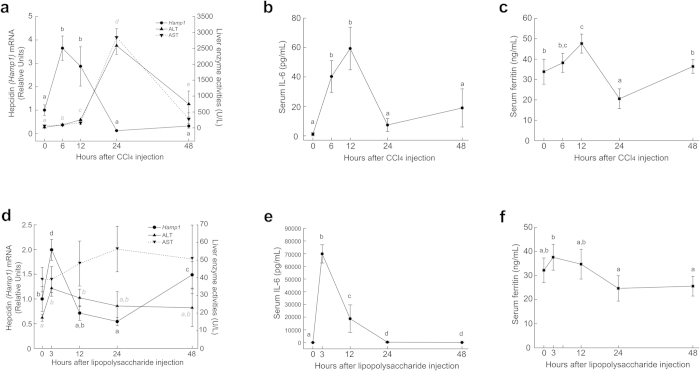
Hepcidin, iron status and transaminases in mouse liver injury models. (**a**) Hepcidin (*Hamp1*) expression and ALT and AST levels were all elevated in the initial stage of injury. Hepcidin expression was 3-fold upregulated (0 h to 12 h) and then repressed. (**b**) Serum IL-6 and (**c**) serum ferritin increased from 0 h to 12 h and then dropped. In lipopolysaccharide-induced liver injury model, similar with CCl_4_ injection, (**d**) hepcidin (*Hamp1*) expression and serum ALT were elevated 3 hours after lipopolysaccharide injection, but AST had no significant change. (**e**) Serum IL-6 dramatically increased from 0 h to 3 h and then dropped. (**f**) Serum ferritin only showed a slight change after lipopolysaccharide injection. Different letters mean significant difference: black letters for hepcidin and grey italic letters for both ALT and AST in (a); black letters for hepcidin and grey italic letters for ALT in (d); *P* < 0.05, ANOVA and Tukey post hoc test (n = 5 in each group).

We hypothesized that increased serum hepcidin may reflected a response to the iron released from liver tissue during disease status. To investigate whether such correlation exists during the process of liver injury, mice were given a single injection of CCl_4_. Both serum ALT and AST levels showed rapid and transient increase from 0 h to 24 h (Fig. 2a). Hepcidin expression showed 3-fold upregulation from 0 h to 12 h. Concurrently, elevated serum IL-6 (Fig. 2b) and serum ferritin (Fig. 2c) were also found to be upregulated from 0h to 12h and then dropped.

Lipopolysaccharide can induce a relative mild liver injury. Mice were given a single injection of lipopolysaccharide. The similar trend was seen in serum ALT when compared to hepcidin expression. Consistent with our cohort study and CCl_4_-induced mouse model, at 3 hour-time point after lipopolysaccharide injection, both hepcidin expression and ALT rapidly increased and then dropped to basal level (Fig. 2d). No significant change was observed in AST. Similar to CCl_4_-induced liver injury model, serum IL-6 drastically elevated at 3 hour-time point after lipopolysaccharide injection (Fig. 2e) while serum ferritin showed a very slight change at 3 hour-time point (Fig. 2f).

## Discussion

Liver enzyme concentrations in human blood including ALT, AST, GGT and bilirubin are routinely tested in recognizing liver diseases such as hepatitis^6^. When hepatocytes are damaged, liver enzymes leak into the circulation and can be detected in the serum. Using population-based association studies, we found positive associations between ALT and serum hepcidin levels in two independent Chinese cohorts consisted of 3455 participants (Table 2, Figure 1). In clinic, both ALT and AST are sensitive markers for detecting liver injury. However, AST is less specific than ALT since AST resides in many other organs, such as heart, skeletal muscle^17^. Therefore, perhaps due to different distributions of these enzymes, only ALT, but not AST and GGT, was found to be associated with serum hepcidin (Table 2). Besides, the fluctuation of the normal ranges of liver enzymes were affected by many factors including liver diseases and genetic factors^18^,^19^.

In our study, despite of the association with serum hepcidin, ALT was also associated with increased serum ferritin and CRP concentrations (Fig. 1). Hepatocytes are the major site of iron storage in the form of ferritin. Increased serum ferritin could result from liver injury^20^ or as an acute phase reactant to inflammatory cytokines^21^. Thus, we hypothesized that increased serum hepcidin reflected a protective response of hepcidin to the iron challenge during liver injury, which it may be in coupled with increased inflammatory parameters (Fig. 1f and 2). Hepcidin secretion can further reduce dietary iron absorption and block iron recycling from macrophages and major organs to ameliorate the iron burden.

Excess iron deposition alone may be not inflammatory^22^. A study reported that HFE C282Y homozygous individuals without concomitant inflammatory liver diseases showed normal transaminases levels^23^. Therefore, the association of elevated serum hepcidin with ALT could be an indicator for the existence of abnormal liver conditions. To functionally validate our hypothesis, two independent mouse liver injury models were applied. Consistently, we observed elevated hepcidin expression and serum IL-6 during the elevation of ALT after CCl_4_ injection (Fig. 2a–c). In a relative mild liver injury mouse model induced by lipopolysaccharide, similar changes of those parameters were observed (Fig. 2d–f).

In contrast, some studies have reported that hepcidin level was reduced in some clinical patients with liver diseases^9^,^10^,^11^. One plausible reason is that the regulation of hepcidin in liver injury is complex, and may vary according to the nature or severity of the injury. For some liver diseases with elevated transaminases, such as chronic hepatitis C^11^ and alcoholic liver diseases^24^, previous studies have shown that serum hepcidin concentration was lower than those found in healthy individuals. A possible explanation is that virus or alcohol may recruit other factors to regulate or even directly regulate hepcidin production^25^,^26^. Other effectors could be growth factors produced during liver regeneration. One functional study has revealed several growth factors including hepatocyte growth factor (HGF) and epidermal growth factor (EGF)^27^ exhibited inhibitory effects on hepcidin expression *in vitro* and *in vivo*. Base on this report (Fig. 2), it is possible that when liver injury is mild, the levels of growth factors are at a relatively low level and the feedback loop of hepcidin is intact. In contrast, when liver damage becomes severe, the high levels of growth factors produced during liver regeneration, which could in turn to disrupt hepcidin regulation. As the result, hepcidin could decrease. One of the major confounding factors of this study is inflammation. But in our cohorts, no association between serum hepcidin and CRP was found in our cohort as previously reported^28^. In one previous study, even serum IL-6, the direct stimulator of hepcidin production^29^, failed to show a correlation with hepcidin^28^.

Iron overload could induce liver injury^7^. Since hepcidin is the master regulator of iron hemostasis, response of hepcidin to iron change is more sensitive and prompt when iron overload co-occurs with liver injury. Based on our observation, the drastically dynamic change of hepcidin is around 3h to 12h while ALT dramatically changed from 12 h to 48 h in CCl_4_-induced liver injury mouse models. We hypothesize that the combination of both ALT and hepcidin may be more faithfully recapitulate the whole dynamic range of the progression of liver injury. Therefore, our findings emphasized the importance of body iron changes during liver injury. Serum hepcidin could be included as potential reference parameters to evaluate liver disease status.

Using hepcidin to predict the initiation of liver injury might be difficult to set up a universal criterion due to both intrinsic and extrinsic heterogeneity of hepcidin expression. One study reported that hepcidin levels are much higher in 40–60 years old females^30^. Since most of the female participants in our cohort studies are above 40 years old, our conclusion cannot be generalized to those females below 40 years old due to the potential physiological differences in their hepcidin concentration and iron status. One limitation of our study is the potential confounders not considered in this study, such as dietary factors associated with transaminase levels. Another limitation is that the mechanisms of hepcidin regulation in liver injury need further investigation in other animal models and hepcidin dysregulated population. A hepcidin knockout mouse model in future study will provide *in vivo* evidence to study the causal relationship between hepcidin and liver injury. Additional population-based studies are warranted to validate these findings in other cohorts and test their potential relevance for the clinical management of hepcidin dysregulation induced liver patients.

## Additional Information

**How to cite this article**: An, P. *et al.* Elevated serum transaminase activities were associated with increased serum levels of iron regulatory hormone hepcidin and hyperferritinemia risk. *Sci. Rep.* **5**, 13106; doi: 10.1038/srep13106 (2015).

## Figures and Tables

**Table 1:** Characteristics of study cohorts.

Trait	Cohort 1				Cohort 2			
	Total	Female	Male	*P* value**	Total	Female	Male	*P* value**
n	1813	1165	648		1642	1042	600	
Age, year	62.12 (9.71)	61.37 (9.64)	63.48 (9.71)	<0.0001	60.72 (8.78)	60.00 (8.75)	61.98 (8.71)	<0.0001
BMI, kg/m^2^	24.76 (3.36)	24.76 (3.41)	24.77 (3.26)	0.47	24.28 (3.17)	24.37 (3.26)	24.13 (3.01)	0.09
Waist Circumference, cm	85.47 (10.40)	83.68 (10.67)	88.67 (9.05)	<0.0001	81.18 (8.69)	80.27 (8.82)	82.78 (8.21)	<0.0001
ALT, IU/L*	12 (10)	12 (10)	12 (10.5)	0.53	14 (9)	13 (9)	14 (10)	<0.0001
AST, IU/L*	25 (11)	25 (11)	25 (10)	0.86	14 (5)	14 (6)	14 (6)	0.0082
GGT, IU/L*	20 (16)	18 (16)	22.5 (16)	<0.0001	18 (15)	16 (13)	23 (21)	<0.0001
IBIL, μmol/L	4.42 (2.26)	4.33 (2.18)	4.58 (2.37)	0.06	4.79 (2.00)	4.57 (1.87)	5.17 (2.14)	<0.0001
DBIL, μmol/L	2.74 (1.31)	2.54 (1.20)	3.08 (1.43)	<0.0001	3.30 (1.32)	3.09 (1.24)	3.67 (1.37)	<0.0001
TBIL, μmol/L	6.98 (3.23)	6.67 (3.06)	7.53 (3.48)	<0.0001	8.09 (3.09)	7.66 (2.86)	8.83 (3.32)	<0.0001
Hepcidin, ng/mL*	25.62 (19.31)	25.33 (18.56)	26.13 (20.15)	0.15	29.94 (25.12)	30.89 (26.03)	29.07 (22.39)	0.01
Ferritin, ng/mL*	245.52 (206.67)	220.66 (184.79)	295.19 (226.24)	<0.0001	195.45 (185.81)	168.22 (164.09)	253.53 (212.68)	<0.0001
Hemoglobin, g/dL	12.75 (1.71)	12.31 (1.45)	13.55 (1.86)	<0.0001	–	–	–	–
CRP, mg/L*	1.23 (1.98)	1.23 (1.98)	1.22 (1.97)	0.83	–	–	–	–
Diabetic status, yes, n, %	955 (52.68)	554 (47.55)	401 (61.88)	<0.0001	160 (9.74)	89 (5.42)	71 (4.32)	0.04
Smoking								
No smoking, n, %	989 (54.55)	732 (62.83)	257 (39.66)	<0.0001	–	–	–	–
Passive smoking, n, %	490 (27.03)	407 (34.94)	83 (12.81)	<0.0001	–	–	–	–
Quitted, n, %	84 (4.63)	4 (0.34)	80 (12.35)	<0.0001	–	–	–	–
Smoking, n, %	243 (13.40)	16 (1.37)	227 (35.03)	<0.0001	–	–	–	–
Drinking								
No drinking, n, %	1533 (84.56)	1122 (96.31)	411 (63.43)	<0.0001	–	–	–	–
Quitted, n, %	38 (2.10)	6 (0.52)	32 (4.94)	<0.0001	–	–	–	–
Drinking, n, %	234 (12.91)	31 (2.66)	203 (31.33)	<0.0001	–	–	–	–

ALT, alanine transaminase; AST, aspartate transaminase; GGT, gamma-glutamyltransferase; CRP, C-reactive protein; DBIL, direct bilirubin; IBIL, indirect bilirubin; TBIL, total bilirubin. Hepcidin (ng/mL) indicates serum hepcidin concentration. Ferritin (ng/mL) indicates serum ferritin.

Data are baseline data and shown as mean (SD).

*Data are shown as median (interquartile range).

**The comparison results between females and males.

**Table 2:** Linear regression of serum hepcidin with serum parameters.

Trait	Cohort 1						Cohort 2					
	Total		Women		Men		Total		Women		Men	
	beta (s.e.)	*P* value	beta (s.e.)	*P* value	beta (s.e.)	*P* value	beta (s.e.)	*P* value	beta (s.e.)	*P* value	beta (s.e.)	*P* value
ALT, IU/L	0.18 (0.01)	<0.0001	0.14 (0.02)	<0.0001	0.23 (0.02)	<0.0001	0.08 (0.02)	<0.0001	0.08 (0.02)	<0.0001	0.07 (0.03)	0.01
AST, IU/L	−0.002 (0.01)	0.56	−0.001 (0.02)	0.67	−0.002 (0.02)	0.73	0.04 (0.01)	<0.0001	0.04 (0.02)	<0.0001	0.03 (0.02)	0.17
GGT, IU/L	0.04 (0.03)	0.57	0.03 (0.03)	0.88	0.05 (0.04)	0.42	−0.004 (0.05)	0.97	0.02 (0.05)	0.60	−0.08 (0.12)	0.34
IBIL, μmol/L	−0.008 (0.003)	0.09	−0.01 (0.004)	0.05	−0.005 (0.005)	0.79	−0.002 (0.003)	0.83	0.005 (0.003)	0.78	−0.009 (0.006)	0.15
DBIL, μmol/L	0.0005 (0.002)	0.89	−0.0002 (0.002)	0.94	0.002 (0.003)	0.82	0.004 (0.002)	0.04	0.004 (0.002)	0.04	0.002 (0.004)	0.27
TBIL, μmol/L	−0.006 (0.004)	0.41	−0.008 (0.005)	0.28	−0.002 (0.007)	0.98	0.003 (0.005)	0.39	0.006 (0.005)	0.22	−0.006 (0.01)	0.63
Ferritin, ng/mL	0.42 (0.18)	0.03	0.41 (0.05)	0.12	0.42 (0.32)	0.16	0.31 (0.21)	0.03	0.39 (0.22)	0.14	0.23 (0.45)	0.13
Hemoglobin, g/dL	0.01 (0.003)	0.002	0.01 (0.004)	0.32	0.01 (0.006)	0.0003						
CRP, mg/L	0.002 (0.003)	0.58	0.003 (0.004)	0.62	0.001 (0.005)	0.64						

ALT, alanine transaminase; AST, aspartate transaminase; GGT, gamma-glutamyltransferase; CRP, C-reactive protein; DBIL, direct bilirubin; IBIL, indirect bilirubin; TBIL, total bilirubin.

Results in Cohort 1 were obtained using linear regression model (hepcidin as an independent variable) adjusted for sex, age, body mass index, smoking habits, drinking categories and diabetic status. Results in Cohort 2 were obtained using linear regression model (hepcidin as an independent variable) adjusted for sex, age, body mass index and diabetic status. Serum hepcidin, ferritin, CRP, ALT, AST, GGT were natural log-transformed, but beta coefficients and standard errors were presented in non-transformed values.

**Table 3:** Associations between transaminases and iron traits.

	Ferritin, ng/mL		Hemoglobin, g/dL		CRP, mg/dL		Hyperferritinemia	
	beta (s.e.)	*P* value	beta (s.e.)	*P* value	beta (s.e.)	*P* value	OR (95% CI)	*P* value
ALT, IU/L	1.69 (0.31)	<0.0001	0.04 (0.007)	<0.0001	0.008 (0.006)	0.0002	1.02 (1.01–1.03)	<0.0001
AST, IU/L	3.44 (0.37)	<0.0001	0.02 (0.006)	0.003	0.02 (0.007)	<0.0001	1.04 (1.03–1.05)	<0.0001
GGT, IU/L	1.27 (0.17)	<0.0001	0.007 (0.003)	0.0008	0.02 (0.003)	<0.0001	1.02 (1.01–1.03)	<0.0001

ALT, alanine transaminase; AST, aspartate transaminase; GGT, gamma-glutamyltransferase; CRP, C-reactive protein.

Results were calculated using baseline data in Cohort 1 (n = 1813). Hyperferritinemia was defined as serum ferritin >200 ng/mL in women and 300 ng/mL in men. Linear and logistic regression models (transaminases as independent variables) were adjusted for sex, age, and body mass index, smoking habits, drinking categories and diabetic status. Ferritin, CRP, ALT, AST, GGT were natural log transformed in analysis, but beta coefficients, standard errors, odds ratio (OR) and 95% confidence interval (CI) were presented in non-transformed values.
